# Clinical value of the correlations of mural coronary artery compression extent with myocardial bridge length and thickness evaluated by 128-slice CT

**DOI:** 10.3892/etm.2012.879

**Published:** 2012-12-28

**Authors:** YU-JUN NIU, XIANG-LIN ZHANG, A-DAN CAO, BING LENG

**Affiliations:** Department of Radiology, The First Affiliated Hospital of Liaoning Medical University, Jinzhou, Liaoning 121001, P.R. China

**Keywords:** mural coronary artery, myocardial bridge, body section radiography, spiral computer, compression extent

## Abstract

This study aimed to investigate the correlations between the detection rate of mural coronary artery (MCA) by 128-slice CT and the MCA compression extent in systole with myocardial bridge (MB) length and thickness. A retrospective analysis was conducted for 580 patients receiving multislicespiral CT coronary angiography (MSCTCA). In addition, the MCA incidence rate and position were detected, and the MB length and thickness in the left anterior descending branch (LAD) and MCA compression extent in systole were measured to compare the differences between MB-MCA length and thickness among the mild, moderate and severe groups. A total of 140 cases of MB-MCA (24.14%) were involved in the study. Among them, 104 cases occurred in the middle segment of the LAD (74.3%), 16 cases (11.4%) occurred in the distal segment of the LAD, 8 cases (5.7%) occurred in the left circumflex-obtuse marginal branch (LCX-OM), 7 cases (5.0%) occurred in the first diagonal branch (1st D), 3 cases (2.1%) in the intermediate branch (M) and 2 cases (1.5%) occurred in the posterior descending branch of the right coronary artery (RCA-PD). The mean length of the MB in the LAD was 21.80±5.98 mm, the mean thickness was 2.15±0.74 mm and the mean compression extent was 38.5±19.6%. Among the different groups, there were no significant difference in MB length (P>0.05) but there were significant differences in MB thickness (P<0.05). In addition, the extent of MCA compression in systole was linearly and positively correlated with MB thickness (r=0.408, P<0.05) but was not correlated with MB length (r=0.076, P>0.05). 128-slice CT coronary angiography (SCTCA) is able to accurately detect MB-MCA and evaluate the correlations of MCA compression extent in systole with MB length and thickness which provides a basis for its clinical use.

## Introduction

The normal coronary artery and its main branch are mostly located in the epicardial adipose tissue. If the original trabecular artery network fails to move outward in the coronary artery development process, any one segment of the coronary artery or its branch is covered by cardiac muscle fiber. This segment of vessel is called a mural coronary artery (MCA), while the bridge-like myocardial fiber bundle covering the artery is called a myocardial bridge (MB). In addition, ‘MB-MCA’ is a complex and is usually called ‘MB’ in the clinic (1). Previously it was understood that MB-MCA was a benign anatomical variant and patients may have no clear symptoms for a long time. At present, a number of patients present with myocardial ischemia and the symptom becomes aggravated, particularly in the case of tiredness, movement and agitation. This may cause stenocardia, ventricular tachycardia, atrioventricular block, acute coronary syndrome, myocardial stunning and even sudden cardiac failure. It is clear that a MB may cause changes to the MCA stressed by it and hemodynamic abnormalities and may cause cardiac events to occur to different extents (2). In the clinic, MB-MCA diagnosis mainly depends on the conventional coronary angiography (CAG) examination. The characteristics of MCA include stenosis in systole and its restoration to the normal level in diastole, presenting a typical ‘milking effect’. However, CAG only demonstrates the persistence of MB-MCA indirectly by observing changes in vessel diameter size at systole and diastole, but it does not directly show the internal and external situations of blood vessel lumen. Only MB-MCA with an marked stenosis may be identified, while a case with superficial stenosis is easily missed. Therefore, the detection rate of CAG is low, only 0.5–16% (3,4). In recent years, the advanced CT technology has improved MB detectability. Certain scholars have used l6- and 64-slice CT to investigate the MB detection rate, and these studies have shown that multiple-slice spiral CT is an effective measure for detecting MB and intuitively shows the thickness, length and imaging characteristics of MB-MCA (5–7). In the present study, a retrospective analysis was conducted of the data from 580 patients with suspected coronary artery lesions receiving 128-slice spiral CT coronary artery angiography (MSCTCA) and the detection rate of MB-MCA was determined. In addition, the left anterior descending branch (LAD) was investigated to observe the correlations of MCA compression extent in systole with MB length and thickness to provide clinical scientific imaging data.

## Materials and methods

### Patients

Data from 580 cases of outpatients and inpatients who received 128-SCTCA in The First Affiliated Hospital of Liaoning Medical University between January, 2011 and December, 2011 were collected. The ages of the patients ranged from 38 to 88 years old and the mean age was 57±12.5 years old. There were 316 male and 264 female cases. Inclusion criteria: patients with clinically suspected coronary heart disease and patient with symptoms of chest tightness, precordial discomfort and effort angina. Disease histories ranged from 1 month to 10 years and written informed consent was obtained from all patients. The present study was approved by the ethics committee of The First Affiliated Hospital of Liaoning Medical University, Jinzhou, China

### Scanning method and parameters

A Definition AS+ scanner (64-detector 128-slice CT; Siemens, Munich, Germany) was used to conduct calcium scoring plain multislicespiral CT (MSCT) scanning. Subsequently, non-ionic contrast agent (iobitridol, 350 mgI/ml, 65–70 ml) was injected with a high pressure injector via the antecubital vein at a rate of 3.5–5.0 ml/sec and 40 ml normal saline was injected to reduce contrast agent dosage and streak artifacts caused by the concentration of the contrast agent being too high in the superior vena cava and right atrium. The aortic root was set as the region of interest (ROI) and the trigger threshold was set as 100 HU. Contrast agent automatic tracking film scanning (timing bolus) was conducted to generate a time-density curve (TDC) and calculate scan delay time. Enhanced scanning of the coronary artery was conducted from the tracheal carina to the diaphragmatic surface of heart under the retrospective electrocardiographically-gated control for 6–11 sec. The scanning parameters were as follows: voltage, 120 kV; current, automatic mA; field of vision (FOV), 160–220 mm; screw pitch, 0.2–0.5; detector collimating value, 64x0.6 mm; acquisition slice thickness, 0.6 mm; rotation speed, 0.33 sec/r; matrix, 512x512; convolution kernel, B26f. In addition, the heart rate was controlled at 75 bpm. For patients with a heart rate >75 bpm, metoprolol (25–50 mg) was provided by sublingual administration 30 min prior to examination to reduce the heart rate.

### Image processing

Circulation software (Siemens) was used to conduct post processing of the optimum images in diastole and systole, and included multiple planar reconstruction (MPR), curve planar reconstruction (CPR), volume rendering technology (VRT), maximum intensity projection (MIP) and Angioview DSA tumbling technology which were used to evaluate the coronary artery. At the 10% R-R interval (the interval between two QRS complexs), a 0–100% R-R interval image was reconstructed. In addition, the Inspace software 4D movie mode was used to observe whether the ‘milking effect’ in systole existed in the MCA segment.

### Image analysis and measurement

**Figure 1.**
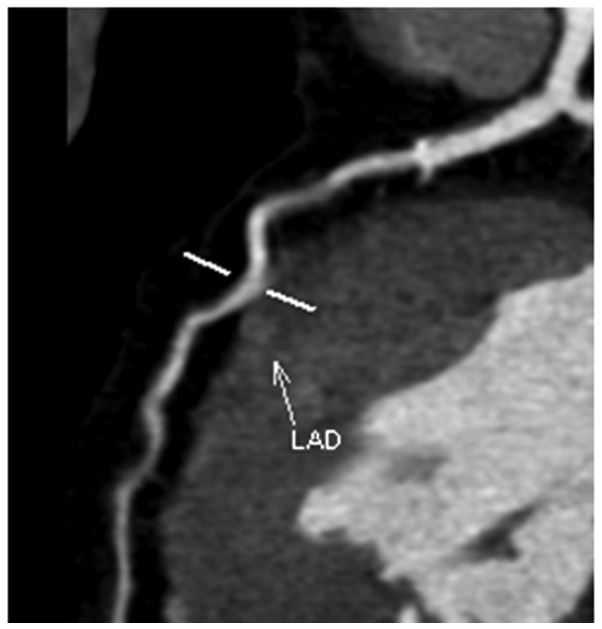
The CPR image shows that the MCA exhibits ‘step up-step down’ or ‘cosine curve’-like changes. CPR, curve planar reconstruction; MCA, mural coronary artery; LAD, left anterior descending branch.

**Figure 2.**
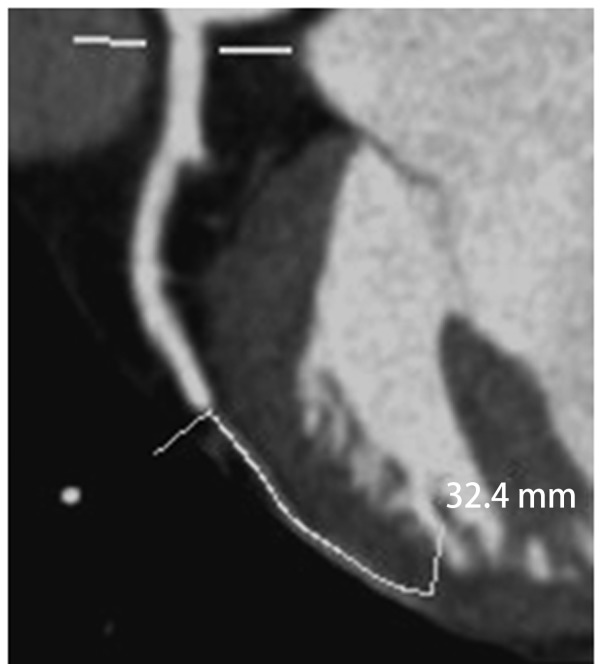
The curved surface length of the MB in the CPR image measured by the scale in the Circulation software. The MCA length in this case is 32.4 mm. MB, myocardial bridge; CPR, curve planar reconstruction; MCA, mural coronary artery.

**Figure 3.**
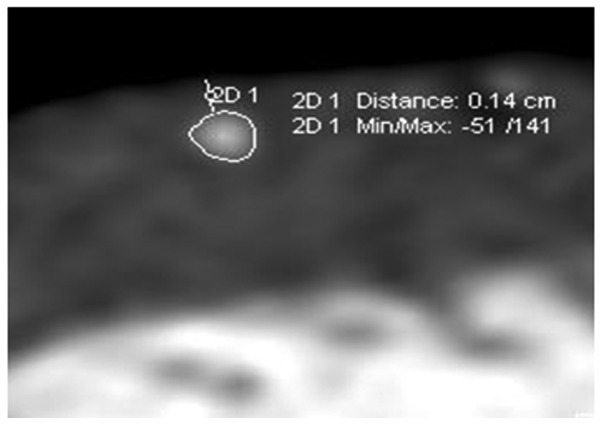
Incisal surface image of MPR (cross section) for measuring the shortest distance from the vascular lateral wall of the MCA to the epimyocardium. The length of the current MB is 1.4 mm. MPR, multiple planar reconstruction; MCA, mural coronary artery; MB, myocardial bridge.

**Figure 4.**
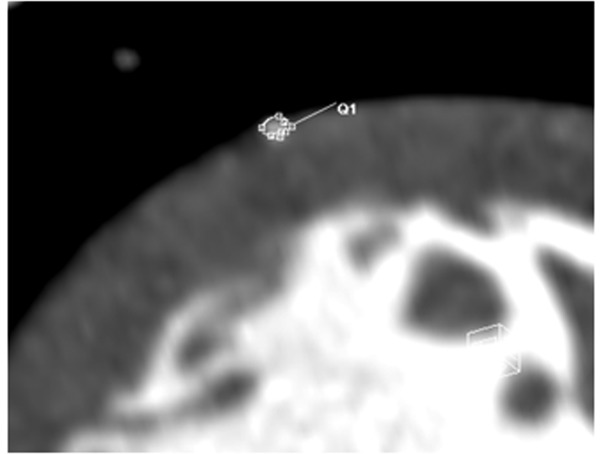
Incisal surface image of MPR (cross section) for measuring the short axis lumen cross-sectional areas of the MCA during diastole and systole phases using the section area method. MPR, multiple planar reconstruction; MCA, mural coronary artery.

The location of the coronary artery with respect to the cardiac muscle was observed. When one segment of the coronary artery was embedded in cardiac muscle or >1/2 the diameter of a segment was surrounded by cardiac muscle or fibrous tissue, while its proximal and distal segments ran in epicardial fat tissues, this segment of coronary artery was evaluated as MCA (8). CPR images of MCA showed ‘step up-step down’ or ‘cosine curve’-like changes on the myocardial surface after running in cardiac muscle for a certain distance (Fig. 1). In addition, MB position, length, depth and compression extent in systole were recorded. i) Position: the coronary artery modification 17-segment model of the American Heart Association (AHA) (9) was used to identify the location. ii) MB length: curved surface length of MCA surrounded by cardiac muscle (Fig. 2); iii) MB thickness: the shortest distance from the vascular wall of the MCA to the myocardial membrane, which was measured at the thickest myocardial cover on MCA cross section (adjust the optimum width and position for observations; Fig. 3); iv) MCA compression extent in systole: the cross-sectional area method was used for evaluation. Rotation was conducted in a vascular CPR image to identify the narrowest position and the short axis lumen areas in diastole and systole (Fig. 4), and the following calculation was performed: MCA compression extent = vascular area of MCA in diastole - vascular area of this segment of MCA in systole / vascular area of MCA in diastole. This process was completed by two physicians with the title of associate chief physician or above.

### Grouping and statistical processing

The cases of LAD with MB were divided into three groups according to the MB-MCA compression extent 3-grade classification method of Noble *et al*(9,10): the mild group (compression extent <50%), the moderate stenosis group (compression extent 50–75%) and the severe stenosis group (compression extent >75%). SPSS 17.0 software (SPSS, Chicago, IL, USA) was used for statistical processing, and measurement data are expressed as the mean ± standard deviation (mean ± SD). The Student’s t-test was used for comparison of measurement data and the Chi-square test was used for comparison of enumerated data. P<0.05 was considered to indicate a statistically significant result.

## Results

### General data

Among the 580 cases who were analyzed, 140 cases presented with MB-MCA and the detection rate was 24.14%. The occurrence sites and constituent ratios are shown in Table I. Among them, 120 cases in which the MB-MCA was located in the LAD, (104 in the middle segment + 16 in the distal segment) were investigated. The MB length ranged between 8 and 46 mm, the mean length was 21.80±5.98 mm, the thickness ranged between 0.7 and 4.4 mm and the mean thickness was 2.15±0.74 mm. The range of the compression extent was between 0 and 69.4% and the mean compression extent was 38.5±19.6%. There were 86 cases of mild stenosis, 26 cases of moderate stenosis and 8 cases of severe stenosis. Among the different groups, no significant difference in MB length was observed (P>0.05) but there were significant differences in MB thickness (P<0.05; Table II).

### Correlation analysis

Pearson’s correlation analysis was conducted for the correlations of MCA compression extent in systole with MCA length and thickness. The extent of MCA compression was linearly correlated with MB thickness (r=0.408, P<0.05) but was unrelated to MB length (r=0.076, P>0.05; Table II, Figs. 3 and 4). This suggests that the thicker the MB, the more marked the MB stress to the MCA, and that the MCA compression extent was not influenced by the MB length.

## Discussion

As a new noninvasive technique, MSCTCA has been widely applied in the clinic, and markedly increases the detection rate of MB. In the present study, the detection rate of 128-slice SCTCA was 24.14% which is comparable to the detection rate (15–85%) of autopsy and higher than that of CAG (0.5–16%). This may be due to the ability of MSCTCA imaging to directly show coronary artery segments that run in cardiac muscle or are partly covered by cardiac muscle. MSCTCA also directly reveals the MB, measures the MB thickness and length, and evaluates the extent of MB lumen compression in systole and the presence of plaques before and after the MCA vessel segment. A ‘milking effect’ may be observed by use of a 4D movie mode. MB-MCA often occurs in the middle and distal segments of the LAD and the coronary artery of left ventricular anterior wall, including the left circumflex-obtuse marginal branch, intermediate branch and diagonal branch. They respectively account for 74.3, 11.4, 5.7, 2.1 and 5.0% of cases, while MB-MCA occurs less frequently in the right coronary artery; the number of right coronary artery cases only accounts for 1.5% of those in the present study, which is almost in agreement with literature values (9–11).

According to the depth (2 mm) by which the MCA is embedded by cardiac muscle, MB is divided into superficial and deep types. The majority of MB cases belong to the superficial type which generally do not cause marked stenosis of the MB segment of the coronary artery in systole, but the deep type may stress and twist vessels, which not only causes MCA stenosis in systole and blood perfusion, but also influences the blood perfusion in the early and medium diastole to cause a clear decrease of coronary flow reserve. In addition, the MCA readily undergoes spasm and secondary atherosclerosis and develops plaque rapture, hemorrhage and thrombosis, thus causing myocardial ischemia and even acute coronary syndrome (ACS). In the present study, 120 cases of patients with MB occurring in the anterior descending branch were grouped according to the MCA compression extent in systole. Comparisons among various groups indicate that that extent of MCA compression correlates with MB thickness but not with MB length, which is in agreement with literature findings (9–13). If the MB thickness is increased, the MCA compression caused by the MB in systole is more evident, and myocardial ischemia symptoms are more severe. Myocardial ischemia is closely correlated with the extent of compression of the MCA, while the latter directly influences the internal diameter of the MCA lumen. According to Poiseuille’s law, blood flow resistance is inversely proportional to the biquadratic of vascular radius. Therefore, MCA compression extent is a main factor causing hemodynamic change. Recent studies also suggest that pressure increases and vortex generation in proximal segments of the MB are the main factors causing atherosis (14,15). As MB-MCA is stressed in systole, long-term compression inevitably causes local vessels to generate high shear stress changes. In addition, electron microscopy and intravascular ultrasound (IVUS) examinations show that endothelial cells of the MB are elongated and almost cover the basal lamina surface. The basal lamina surface is covered with microvilli. While the endothelial cells of the coronary artery in the proximal MB mostly present flap or oval shapes, the cell surfaces present rough worm-eaten-like defects. The vessels in the distal segment of the MB are in a relatively low pressure state and the surfaces present fewer worm-eaten-like defects, which is possibly associated with the anti-atherosclerosis effect of high shear stress. As high shear stress usually induces endothelial cells of the MCA to express vascular relaxing factor, growth inhibiting factor, fibrinolysis substance and antioxidant, and inhibits the expression of vascular contraction factor, growth factor, inflammatory mediator and adhesion factor, it cannot easily damage endothelial cells and is detrimental for cellular proliferation, lipid uptake and blood cell adhesion. Thus, high shear stress has an anti-AS effect. In addition, the position of the MB has a certain influence on myocardial ischemia. If it is closer to the coronary sinus, particularly for thicker MB, its stress to vessels is marked (14–16).

In summary, 128-slice SCTCA directly reveals the positions of MCA and cardiac muscle, but also dynamically evaluates the presence of MCA-MB and dynamically shows changes of MCA lumen size by exhibiting the ‘milking effect’ with a 4D movie mode. In particular, 128-slice SCTCA is more sensitive for the detection of superficial MBs. In addition, 128-slice SCTCA further enables the determination of MB thickness and length and compression extent in systole. Therefore, 128-slice SCTCA may provide extensive imaging information for the preoperative evaluation of surgical muscle bridge lysis and thereby guide surgery. Furthermore, 128-slice SCTCA may provide a basis for the preoperative judgement of MB position, thickness, length and compression extent in interventional treatment and the accurate and effective selections of stent type and length, which increases the treatment success rate (17–20). However, this study also shows certain shortcomings (the diagnosis results are not comparable with those of the pathological gold standard and the technique is unable to calculate to high sensitivity and specificity) due to the spatio-temporal resolution limitations of MSCT and the influence of motion artifacts on compression extent accuracy. Therefore, it is necessary to conduct further modifications and studies.

## Figures and Tables

**Table I.** MB-MCA locations and constituent ratios.

Incidence	Middle segment of LAD	Distal segment of LAD	LCX-OM	1st D	Intermediate branch	RCA-PD	Total
No. of cases	104	16	8	7	3	2	140
Constituent ratios (%)	74.3%	11.4%	5.7%	5.0%	2.1%	1.5%	100%

MB, myocardial bridge; MCA, mural coronary artery; LAD, left anterior descending branch; LCX-OM, left circumflex-obtuse marginal branch; 1st D, first diagonal; RCA-PD, posterior descending branch of the right coronary artery.

**Table II.** Correlations of the MCA systolic compression extent with the length and thickness of the MB.

Muscle bridge	Mild stenosis group (n=86)	Moderate stenosis group (n=26)	Severe stenosis group (n=8)	F	P-value
Length (mm)	21.18±5.85	23.91±5.48	21.81±5.97	2.139	0.122
Thickness (mm)	1.94±0.63	2.51±0.71	3.24±0.76	19.213	0.000

MB, myocardial bridge; MCA, mural coronary artery.
